# ASO Author Reflections: Intraoperative Radiation Therapy (IORT): Near Dead in the USA

**DOI:** 10.1245/s10434-025-17611-3

**Published:** 2025-06-05

**Authors:** Melvin J. Silverstein, Brian Kim, Kevin Lin, Shane Lloyd

**Affiliations:** 1 Department of Surgery, Keck School of Medicine, University of Southern California, Los Angeles, CA USA; 2 Department of Surgery, Hoag Memorial Hospital Presbyterian, Newport Beach, CA USA; 3 Department of Radiation Oncology, Hoag Memorial Hospital Presbyterian, Newport Beach, CA USA

## Past

We write these *Author Reflections* with pride for what we have done as a team over the last 15 years and with sadness as we end our intraoperative radiation therapy (IORT) program. In this issue of the *Annals*, we report the outcome for 1828 early-stage breast cancers treated within our IORT program and the reasons why we discontinued the program.^1^

At the end of the last century, IORT was an exciting newcomer to the breast cancer treatment armamentarium, a great idea! It delivered the entire course of radiation therapy in the operating room during surgical excision of the primary tumor, eliminating 5–6 weeks of whole-breast radiation therapy (WBRT), which was standard at the time.

IORT treated the tumor bed and approximately a 1-cm margin. It was essentially a larger lumpectomy without the deformity created by taking an additional centimeter of tissue in every direction. Since it did not treat the whole breast, it was not likely to be as good as WBRT at decreasing local recurrence, but hopefully, it would be almost as good and that might be acceptable. “Almost as good” would be offset by the many benefits of IORT.

IORT was less expensive and took less time (one dose versus many). There was less pain. It was cosmetically superior, particularly for the augmented patient, and there was no breast shrinkage. IORT was much better for patients living at any distance from the radiation therapy center. There was no travel back and forth, so it was “green”, less gas, less pollution. Busy people got back to their normal life quicker. It was better for patients with challenging conditions such as those who were wheelchair bound, had neurologic diseases or autism, etc. It was better for the unhoused, the unreliable patient, and the incarcerated. It was better for non-English-speaking patients. There was less hospital exposure, so less risk of airborne respiratory illnesses, a blessing during the coronavirus disease 2019 (COVID-19) pandemic. Finally, there was less radiation dose to the skin, heart, and lungs, with virtually no damage to surrounding organs.

While IORT  was extremely convenient for the patient, this  was not the case for the medical professionals and the hospital. Radiation oncology needs a team, consisting of a physicist, a technician, and a physician, in the operating room for about an hour. The breast surgeon needs approximately 20 extra minutes to prepare the excision cavity for IORT. The schedulers must find a time to get everyone together in the operating room (OR). All must then deal with the inevitable delays of the OR schedule. And finally, the hospital loses the technical revenue associated with multiple treatments in their radiation therapy unit.

## Present

The second and third decades of this century brought improvements in radiation oncology. Hypofractionation shortened the course of treatment. There were 15-day courses of WBRT and then shorter, 5-day courses of whole-breast or partial-breast irradiation (APBI). With new shorter effective alternatives, the benefits of IORT began to diminish.

Recently, long-term results of IORT became available. Two prospective randomized IORT trials have been reported. The ELIOT trial, which used electrons, initially reported 4.4% local recurrence for patients treated with IORT compared with 1.4% for WBRT at 5 years. But, at 15 years, they reported 12.6% local recurrence for patients treated with IORT compared with 2.4% for WBRT.^2^

The TARGIT-A trial used 50-kV photons and a risk-adapted approach, meaning they added WBRT when poor prognostic factors were found on final histopathology. This happened often, about 20–30% of the time, depending upon which of their datasets is analyzed. TARGIT-A divided their reports into two groups: those who received IORT at the time of initial surgery (pre-pathology) and those who received IORT at a second surgery (post-pathology). The pre-pathology group reported 2.1% local recurrence at 5 years for patients treated with IORT compared with 1.1% for WBRT.^3^ The post-pathology group reported 5.4% local recurrence for patients treated with IORT compared with 1.7% for WBRT.^4^ There have been many questions about the TARGIT-A data and multiple re-analyses. The TARGIT-A group never published recurrence data for the subgroup who met their criteria and received IORT alone, making it challenging to interpret.

The results of IORT have been acceptable in Europe, and it continues to be popular there, likely because many countries have nationalized healthcare and can benefit from single-dose treatment. In the USA, we have a fee-for-service system for most patients, although that is changing. From the beginning, IORT has been reimbursed poorly in the USA, or not at all. When it pays, it pays for a single treatment rather than 15 or 30 treatments. Financially, there is little incentive for the hospital or the doctors to perform IORT. This is likely the reason why only about 100 hospitals in the USA ever offered IORT.

So, up to this point, we had a new, extremely convenient form of breast radiation therapy that patents loved, and doctors and hospitals accepted for the benefit of the patients. As long-term results began to accrue, it became clear that the local recurrence rate for IORT was higher when compared with more the current, standard forms of radiation therapy, but it was still relatively low, around 4–5% at 5 years. On a positive note, the long-term data showed no difference in overall or disease-specific survival at 15 years.^2^ So, in exchange for extreme convenience, the patient got a higher local recurrence rate but with no impact on survival.

For about 20 years, IORT walked a fine line in the USA, with only marginal acceptance and minimal use. Then a critical position paper by the most important group of radiation oncologists in the world was published: the 2024 American Society of Radiation Oncology (ASTRO) Consensus on Accelerated Partial Breast Irradiation.^5^ ASTRO recommended against all forms of IORT unless they were within a clinical trial or multicenter registry. ASTRO was strongly influenced by the 12.6% local recurrence rate of the ELIOT trial at 15-years and the difficulty of interpreting the TARGIT-A data versus the extremely low local recurrence rates of current, more standard forms of radiation therapy.

Our IORT group met twice a year for the duration of the program. We reviewed our data and all additional IORT data published throughout the world. Every year, we agreed to continue offering IORT. But ASTRO’s 2024 publication caused a detailed and thoughtful re-evaluation of our program. In the end, our radiation oncologists felt they could not continue to offer IORT without the support of their society. Our group regretfully agreed. In June 2024, we did our last IORT case.

## Future

So that is that. In June, we pushed the IORT machine into an alcove in one of the long corridors of the operating room. Then, a few months later, it went to the basement of the hospital, and at some point, it will go into the recycling bin. And that is how it goes. IORT is near dead in the USA.
